# Safety and efficacy of continuous renal replacement therapy for children less than 10 kg using standard adult machines

**DOI:** 10.1007/s00431-023-05007-y

**Published:** 2023-05-26

**Authors:** Manju Kedarnath, Emma C. Alexander, Akash Deep

**Affiliations:** 1 Paediatric Intensive Care Unit, King’s College Hospital NHS Foundation Trust, Denmark Hill, London, UK; 2 Department of Women and Children’s Health, School of Life Course Sciences, King’s College London, London, UK

**Keywords:** Kidney replacement therapy, Acute liver failure, Acute kidney injury, Infants, Neonates, Circuit life

## Abstract

Continuous Renal Replacement Therapy (CRRT) machines are used off-label in patients less than 20 kg. Infant and neonates-dedicated CRRT machines are making their way into current practice, but these machines are available only in select centres. This study assesses the safety and efficacy of CRRT using adult CRRT machines in children ≤ 10 kg and to determines the factors affecting the circuit life in these children. Design: Retrospective cohort study of children ≤ 10 kg who received CRRT (January 2010-January 2018) at a PICU in a tertiary care centre in London, UK. Primary diagnosis, markers for illness severity, CRRT characteristics, length of PICU admission and survival to PICU discharge were collected. Descriptive analysis compared survivors and non-survivors. A subgroup analysis compared children ≤ 5 kg to children 5–10 kg. Fifty-one patients ≤ 10 kg received 10,328 h of CRRT, with median weight of 5 kg. 52.94% survived to hospital discharge. Median circuit life was 44 h (IQR 24–68). Bleeding episodes occurred with 6.7% of sessions and hypotension for 11.9%. Analysis of efficacy showed a reduction in fluid overload at 48 h (*P* = 0.0002) and serum creatinine at 24 and 48 h (*P* = 0.001). Blood priming was deemed to be safe as serum potassium decreased at 4 h (*P* = 0.005); there was no significant change in serum calcium. Survivors had a lower PIM2 score at PICU admission (*P* < 0.001) and had longer PICU length of stay (*P* < 0.001).

*Conclusion*: Pending neonatal and infant dedicated CRRT machines, CRRT can be safely and effectively applied to children weighing ≤ 10 kg using adult-sized CRRT machines.

**Table:** 

**What is Known:** *• Continuous Renal Replacement Therapy can be used for a variety of renal and non-renal indications to improve outcomes for children in the paediatric intensive care unit. These include, persistent oliguria, fluid overload, hyperkalaemia, metabolic acidosis, hyperlactatemia, hyperammonaemia, and hepatic encephalopathy.* *• Young children ≤ 10 kg are most often treated using standard adult machines, off-label. This potentially places them at risk of side effects due to high extracorporeal circuit volumes, relatively higher blood flows, and difficulty in achieving vascular access.*	
**What is New:** *• This study found that standard adult machines were effective in reducing fluid overload and creatinine in children ≤ 10 kg. This study also assessed safety of blood priming in this group and found no evidence of an acute fall in haemoglobin or calcium, and a fall in serum potassium by a median of 0.3 mmol/L. The frequency of bleeding episodes was 6.7%, and hypotension requiring vasopressors or fluid resuscitation occurred with 11.9% of treatment sessions.* *• These findings suggest that adult CRRT machines are sufficiently safe and efficacious for routine use in PICU for children ≤ 10 kg and suggest that further research is undertaken, regarding the routine rollout of dedicated machines.*

**What is Known:**

*• Continuous Renal Replacement Therapy can be used for a variety of renal and non-renal indications to improve outcomes for children in the paediatric intensive care unit. These include, persistent oliguria, fluid overload, hyperkalaemia, metabolic acidosis, hyperlactatemia, hyperammonaemia, and hepatic encephalopathy.*

*• Young children ≤ 10 kg are most often treated using standard adult machines, off-label. This potentially places them at risk of side effects due to high extracorporeal circuit volumes, relatively higher blood flows, and difficulty in achieving vascular access.*

**What is New:**

*• This study found that standard adult machines were effective in reducing fluid overload and creatinine in children ≤ 10 kg. This study also assessed safety of blood priming in this group and found no evidence of an acute fall in haemoglobin or calcium, and a fall in serum potassium by a median of 0.3 mmol/L. The frequency of bleeding episodes was 6.7%, and hypotension requiring vasopressors or fluid resuscitation occurred with 11.9% of treatment sessions.*

*• These findings suggest that adult CRRT machines are sufficiently safe and efficacious for routine use in PICU for children ≤ 10 kg and suggest that further research is undertaken, regarding the routine rollout of dedicated machines.*

## Introduction

Acute kidney injury occurs in up to a quarter of children admitted to Paediatric Intensive Care Units (PICUs) and is independently associated with increased mortality [1]. Continuous Renal Replacement Therapy (CRRT) is therefore a supportive therapy that has the potential to significantly reduce mortality in PICU. CRRT is advantageous as a treatment modality in the critically ill child because lower flow rates (compared to intermittent replacement) reduce the risk of hypotension and raised intracranial pressure, which would be inadvisable in unstable patients in multi-organ failure [2]. A large study of 129,809 PICU admissions in the UK found that 2.9% of all patients admitted to PICU received renal replacement therapy, of which just over half (50.3%) received CRRT, with others receiving peritoneal dialysis, a combination of both modalities, or intermittent haemodialysis [3].

Indications for CRRT have widened in recent years beyond acute kidney injury, fluid overload and electrolyte imbalances [4], to include a range non-renal indications including metabolic abnormalities, hyperammonaemia > 150 μmol/L and Grade III/IV hepatic encephalopathy in children with liver failure [5, 6], drug toxicity, and sepsis [7]. However, much of the evidence for CRRT is based on observations in adults, for whom standard machines were developed.

Where CRRT is applied to children, most machines are used off label for patients ≤ 20 kg [8]. The need for appropriate treatment modalities in children ≤ 10 kg is pressing. A prospective cohort analysis of children who received CRRT showed that children ≤ 10 kg had lower survival rates than children > 10 kg [9]. However, there is no universally appropriate alternative for these small children. Peritoneal dialysis is not appropriate for all neonates, such as those who may have required abdominal surgery, and peritoneal catheters are more likely to fail if they require immediate use, or for infants under one month of age [10, 11]. These restrictions limit its use for many children ≤ 10 kg. However, using adult CRRT machines in these smaller children also carries a theoretical risk, because of higher extracorporeal circuit volumes relative to patient blood volume. Circuits are ordinarily primed with blood to prevent sudden dilution after commencing treatment, with priming volume ranging between 60 ml and > 250 ml [12]. This is challenging for children under 10 kg, with total blood volume for a 3 kg new-born being around 240 ml, and 800 ml for a 10 kg infant [13]. Using more than 10% of the child’s circulating blood volume to prime a circuit may risk hypotension or anaemia [14]. Priming also runs the risk of electrolyte changes [15]. In smaller children, blood flows are relatively higher, and there are concerns about the margin of error in maintaining accurate fluid balance [16]. Finally, obtaining vascular access can be difficult which adds to the technical challenges of providing CRRT to smaller children.

Through several advancements in the field of critical care nephrology, dedicated neonatal/infant machines are being manufactured which can be safely used in smaller children [14]. However, these machines are not yet universally available. Therefore, the majority of CRRT programs continue to use standard CRRT machines for adults in the smallest children. This study aims to describe the safety and efficacy of adult-based machines in the use of CRRT in children ≤ 10 kg.

## Materials and methods

### Study population

King’s College Hospital, London is a tertiary level centre for paediatric intensive care, with a large supra-regional center for liver referrals in the United Kingdom that operates one of the largest liver transplantation programs in Europe, because of which, the use of CRRT is high. Data were collected retrospectively for all children weighing ≤ 10 kg who received CRRT from January 2010-January 2018.

### Data collection

Medical records, laboratory data, and observation charts were reviewed for all patients. Patient characteristics including age, gender, and weight were recorded. Clinical observations and laboratory data were collected from the unit’s Clinical Information System, Metavision. Paediatric Index of Mortality (PIM) 2 was calculated for each patient [17]. Duration of Paediatric Intensive Care Unit (PICU) stay, indication for CRRT, underlying diagnosis, time from PICU admission to initiation of CRRT and survival on hospital discharge were collected.

On initiation of CRRT, baseline characteristics collected were: indication for initiation of CRRT, laboratory results (serum creatinine, urea, lactate, bicarbonate and base excess), duration of CRRT, vascular access (size and location), use of blood priming, use of anticoagulant, vasopressor requirement, oxygen and ventilation pressure requirement, CRRT dose, maximum blood flow achieved, circuit life and reason for circuit change. The daily percentage fluid overload was calculated using the Goldstein formula [18].

Indications for CRRT initiation were classified: acute kidney injury (AKI), acute liver failure, hyperammonemia greater than 150 μmol/L, lactate greater than 2 mmol/L not responding to fluids or vasopressors, and fluid overload greater than 10%. Initiating CRRT is a clinical decision and no single indication is considered absolute.

We looked at markers of efficacy and safety of CRRT targeted to issues related to infants and children < 10 kg. Efficacy markers were median filter life, serum creatinine and percentage drop in FO after 24 and 48 h of CRRT initiation. Safety markers were change in serum haemoglobin, potassium and calcium recorded after 4 h of initiating CRRT as markers of potential complications of blood priming, as well as new bleeding episodes or hypotension on CRRT initiation.

#### CRRT

The duty consultant paediatric intensivist determined the requirement for CRRT. CRRT was commenced according to a local protocol with predilution Continuous Veno-Venous Hemofiltration (CVVH) to achieve required electrolyte and fluid homeostasis. All children had ultrasound-guided venous access via a high-flow double lumen catheter (“Vascath”; Gambro, Stockholm, Sweden) placed either in the internal jugular, femoral or subclavian vein. The machine used for CRRT was “Aquarius” (Nikkiso Europe GmbH, Hannover, Germany). Prostacyclin (Flolan, GlaxoSmithKline UK) was our default anticoagulant (4–8 ng/kg/min). To achieve adequate toxin/solute clearance, CRRT dose was initiated at 60 mL/kg/hr and sequentially increased to a maximum of 100 mL/kg/hr. This strategy was applied as most of our patients had liver failure with hyperammonaemia; inborn errors of metabolism; or sepsis/AKI/fluid overload (FO) in those with liver failure. In practice, indications of AKI/FO in the absence of liver failure or inborn error of metabolism do not warrant such high dose and traditional doses at 30–40 mL/kg/hour can be used which may allow lower blood flow rates, anticoagulation usage and smaller vascular catheters. The sizes of the catheters used were predefined according to body weight. All children less than 10 kg were filtered using Aquamax HF03, a smaller filter size composed of polyethersulphone with a membrane surface area of 0.3m^2^, priming volume of 32 ml and membrane cut-off of 55Kda applied with Aqualine-S, a relatively smaller circuit with priming volume of 61 ml as compared to standard adult-sized circuit (Aqualine) with a priming volume of 100 ml. Predilution was incorporated in all filtration episodes using “Accusol 35”, a lactate-free electrolyte solution.

###  Statistical analysis

Statistical data were analysed with Stata Version 16 and SPSS Version 27. Descriptive analyses were performed to determine differences between survivors and non-survivors, and children weighing < 5 kg with those 5–10 kg. All data were analysed with the Shapiro-Wilk test for normality. Normally distributed continuous variables were compared using the Student’s *t*-test and reported as mean (± SD). Non-normally distributed variables were analysed using the Mann-Whitney test as median (25% IQR, 75% IQR). Categorical variables were analysed with Chi Squared/Fisher’s exact test. For descriptive statistics, a *P* value < 0.05 was considered statistically significant. Patients with missing data were excluded from analyses if applicable. Univariate analysis identified association between patient characteristics and survival. Multivariate logistic regression analysis was performed to control for confounding variables. Based on clinical and statistical significance from the univariate analysis, variables with a *P* value > 0.2 were eliminated. Kaplan Meyer analysis was used to analyse the cumulative circuit life. Patient and circuit related factors affecting the circuit life were also determined.

### Ethical approval

Since this was a retrospective analysis of previously collected routine clinical data, formal ethical approval was not sought. However, this project was registered as a service improvement project at King’s College Hospital, London.

## Results

From January 2010 to January 2018, 51 patients ≤ 10 kg met the inclusion criteria of receiving CRRT while being managed on the PICU. Twenty-seven patients survived to discharge from PICU (52.9%). Median weight was 5 kg, with the smallest child weighing 1.75 kg (catheterised percutaneously with a 6.5 French catheter in the internal jugular vein). Median age was 123 days. Males constituted 50.9% of the cohort. All patients were treated with the CVVH modality, receiving a total 10,328 h of treatment. The most common site for vascular access was the internal jugular (76.5%) followed by femoral (15.7%) and subclavian veins (7.8%). The most common size for vascular access was 6.5F (82.4%). Baseline characteristics are in Table 1.

**Table 1:** Primary characteristics of children - survivors and non-survivors, and total cohort

	**Survivors (n = 27)**	**Non-Survivors(n = 24)**	**Total**
Weight (kg)	6.48 [3.88, 10.00]	4.00 [3.32, 5.28]	5.00 [3.50, 7.06]
Age (d)	194.00 [16.00, 395.00]	25.50 [9.50, 172.50]	123.00 [11.00,294.00]
Proportion aged 28 days or below at initiation (n, %)	8 (29.6%)	13 (54.2%)	21 (41.2%)
PICU Length of Stay (d)	24.00 [13.00, 33.00]	7.00 [2.00, 12.00]	13.00 [6.00,26.00]
PICU hours prior to CRRT initiation	20.00 [5.00, 62.50]	13.50 [6.00,41.25]	15.00 [6.00,49.50]
PIM2 score at ICU admission	19.20 [3.91, 45.90]	55.48 [40.08, 78.95]	39.21 [14.97, 64.73]
Paw at CRRT initiation	14.00 [10.00,24.00]	20.00 [13.00,25.50]	17.00 [10.00,24.00]
Overall balance at CRRT start (L)	0.34 [0.16, 0.75]	0.41 [0.14, 0.73]	0.35 [0.15, 0.75]
FO% at CRRT start	7.17 [2.47,12.11]	10.01 [3.99,14.73]	7.71 [2.64,13.17]
Blood flow rate (ml/kg/min)	8.80 [7.69,12.50]	13.60 [7.71,17.83]	10.80 [7.69,14.90]
CRRT dose (ml/kg/hr)	60.00 [51.50,79.00]	60.00 [47.00,66.00]	60.00 [51.00,67.00]
Hours of CRRT incl downtime	138.00 [71.00,407.00]	67.00 [20.00,218.50]	90.00 [42.00,282.00]
Hours of CRRT downtime	3.00 [1.00,13.00]	2.50 [0.00,5.50]	3.00 [0.00, 11.00]
Hours of CRRT effective	120.00 [70.00,384.00]	64.50 [16.00,210.00]	88.00 [42.00,269.00]
Filter number	3.00 [2.00,8.00]	2.00 [1.00,3.50]	2.00 [1.00,5.00]
FiO2 req at CRRT initiation	52.89 [20.04]	72.62 [24.67]	62.18 [24.24]
Maximum Blood flow (ml/min)	64.59 [26.79]	54.17 [20.81	59.69 [24.49]
Mean filter life per individual	43.48 [20.58]	37.25 [22.92]	40.55 [21.72]

Median [p25,p75], n (%) or Mean [SD]

Patients spent a median of 15 h in the PICU prior to receiving CRRT (Table 1). The majority of patients (*n* = 34, 66.7%) had multiple indications for starting CRRT. The most common indications were acute kidney injury (*n* = 34; 66.7%), acute liver failure (*n* = 27; 52.9%), fluid overload > 10% (*n* = 21; 41.2%), and high lactate (*n* = 19; 37.3%).

### Efficacy

A total of 223 circuits were used for a total duration of 10,328 h of CRRT. The median (IQR) circuit life was 44 h (24,68). The most common reason for circuit change was elective change (for 54.7% of filters) (Table 2). The median blood flow rate (ml/kg/min) was 10.8 (IQR 7.7-14.9).

**Table 2:** Reason for circuit change (ANOVA analysis)

**Reasons for Circuit change**	**N (%)**	**Circuit life Mean ± SD**	***P*** **value**
Elective termination or Change	122 (54.71)	55.30 ± 25.67	< 0.01
Vascath Related problems	23 (10.31)	26.65 ± 21.54	
Filter/Circuit related problems	71 (31.84)	34.98 ± 23.90	
Multiple reasons of filter clot	7 (3.14)	28 ± 18.25	

Serum creatinine decreased after 24 and 48 h of CRRT treatment. In 22 patients who were fluid overloaded (FO > 10%) there was a clinical and statistically significant decrease in fluid overload (Table 3).

**Table 3:** Serial progression markers

***Blood priming markers***					
Marker	**CRRT (0 h)**		**CRRT (4 h)**		***P* value**
Serum hemoglobin, mmol/L^a^	91	(72, 121)	97	(73, 117)	0.497
Serum potassium, mmol/L^a^	4.2	(3.3, 5.8)	3.9	(3.3, 4.4)	**0.005**
Serum calcium, mmol/L^a^	2.14	(1.77, 2.79)	2.11	(1.75, 2.43)	0.126

***CRRT Efficacy markers***					
**Marker**	**Value**		***P value*** **(vs CRRT 0 h)**		
Serum creatinine, mmol/L^a^					
CRRT (0 h) (n = 41) CRRT (24 h) (n = 39) CRRT (48 h) (n = 38)	53 (23,263) 45 (22,93) 41.5 (20,75)		- 0.001 0.001		
Fluid overload, %^b^					
CRRT (0 h) (n = 22) CRRT (48 h) (n = 16)	13.91 (12.10,16.56) 1.93 (-3.01,4.79)		- 0.0002		

Bold denotes *p*<0.05

^a^Median (IQR 25%, 75%)

^b^Mean ± SD

### Safety

Serial progression in markers of CRRT safety (related to CRRT initiation, vascular access insertion, blood priming and anticoagulation) and efficacy were collected from the clinical data. In terms of safety, there was no evidence of an acute fall in serum haemoglobin in our cohort. Despite blood priming, serum potassium significantly decreased (from median 4.2 mmol/L to 3.9 mmol/L, *P* = 0.005), and serum calcium did not change (Table 3). In terms of complications, bleeding episodes either during Vascath insertion or post-CRRT initiation were minimal (6.7%) and newly developed hypotension requiring fluid resuscitation or vasopressor support post-CRRT initiation was linked to 11.9% of the treatment sessions.

### Comparing children undergoing CRRT weighing < 5 kg versus those 5–10 kg

We performed a subgroup analysis to identify differences in baseline demographics, CRRT circuit characteristics and survival in children weighing 5–10 kg and smaller children weighing ≤ 5 kg (Table 4). Survival was lower in children weighing < 5 kg (36.0% vs 69.2%, *P* = 0.025). Indications for CRRT and primary diagnosis were not significantly different between the two groups except that a lower proportion of children ≤ 5 kg had a primary diagnosis of chronic liver disease/biliary atresia.

**Table 4:** Comparison of characteristics of CRRT in children according to weight (< 5 kg versus 5–10 kg)

**Data**	** ≤ 5 kg (N = 25)**		**5–10 kg (N = 26)**		***P*** **value**
Key indication for CRRT, *n* (%)					
Renal failure ALF High ammonia High lactate Fluid overload Multiple reasons	19 (76) 12 (48) 8 (32) 8 (32) 13 (52) 18 (72)		15 (57.7) 15 (57.7) 5 (19.2) 11 (42.3) 8 (30.8) 16 (61.5)		0.166 0.488 0.296 0.447 0.124 0.428
Primary diagnosis, *n* (%)					
Acute liver failure	13 (52)		11 (42.3)		0.488
Chronic liver disease/biliary atresia	1 (4)		11 (42.3)		**0.001**
Sepsis Inborn error of metabolism Other *Some patients had multiple primary diagnoses	6 (24) 6 (24) 12 (48)		6 (23.1) 8 (30.8) 8 (30.8)		0.938 0.588 0.208
CRRT catheter site, n (%)					
Femoral vein Internal jugular vein Subclavian vein	3 (12) 20 (80) 2 (8)		5 (19.23) 19 (73.08) 2 (7.69)		0.883
CRRT catheter size, n (%)					
6F 6.5F 8F 10F	1 (3.85) 24 (96) 0 0		0 18 (69.2) 7 (26.9) 1 (4)		**0.008**
Blood flow rate (mL/kg/min)*	14.29	(11.36,17.80)	8.60	(7.14,10.80)	** < 0.01**
Filters per patient*	5	(3,16)	8	(5,21)	< **0.01**
Reason for circuit change, n (%)					
Elective change Filter issue Vascath issue Multiple reasons	55 26 5 4	(61.11%) (28.89%) (5.56%) (4.44%)	67 45 18 3	(50.38%) (33.83%) (13.53%.) (2.26%)	0.128
Filter Life (hrs.)*	52	(35,71)	38	(19,64)	**0.002**
CRRT dose*	60	(52,80)	60	(45,66)	0.138
Survival (n (%))	9	(36.0)	18 (69.2)		**0.025**

Bold denotes *p*<0.05

^*^Median (IQR 25%, 75%); N (%)

**Fig. 1:**
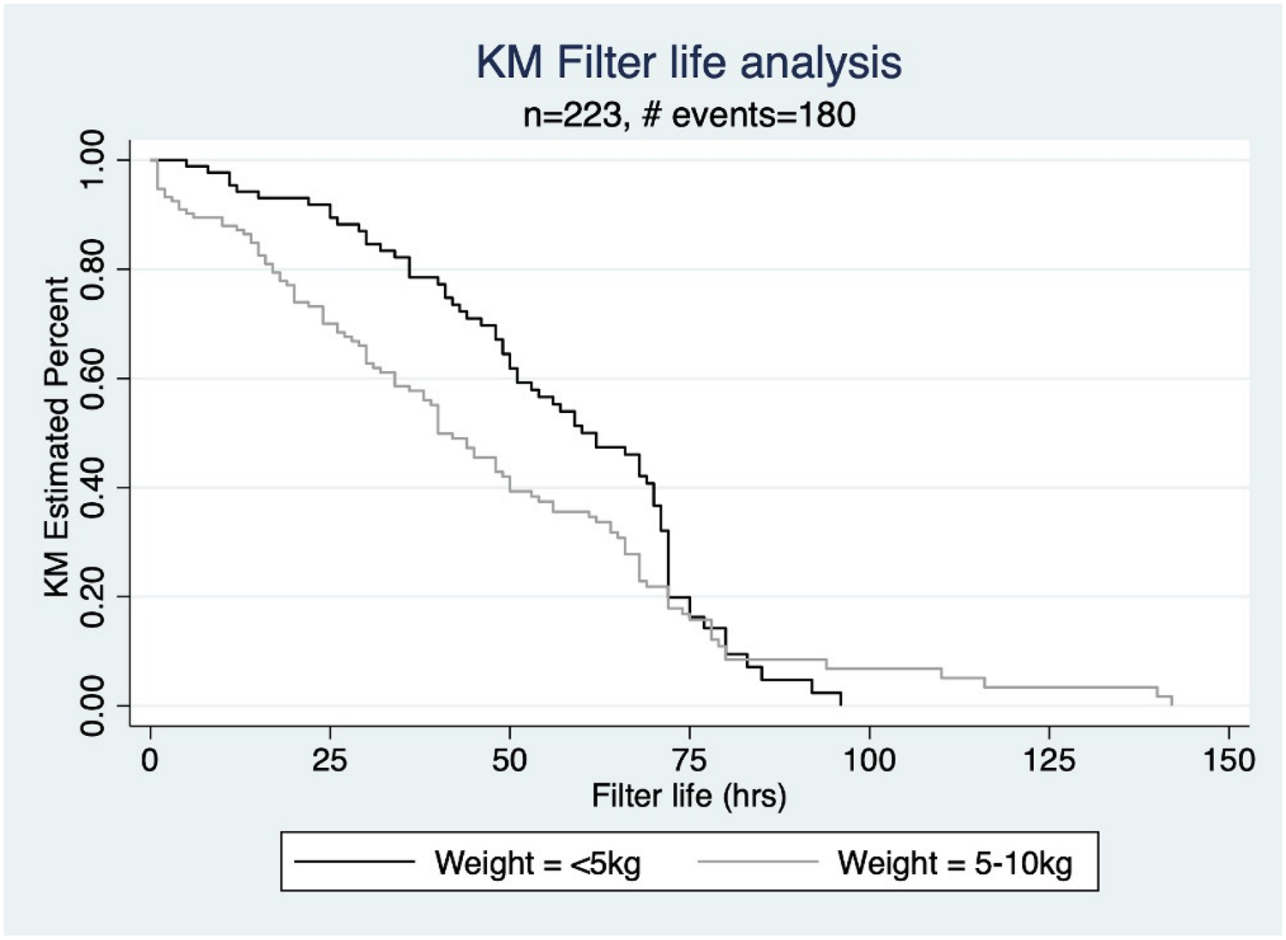
Weight based comparison of filter-life in infants < 5 kg versus 5–10 kg, P = 0.0532

Ninety circuits were used for children weighing ≤ 5 kg, and 133 circuits for children weighing 5–10 kg. Vascular access sites were similar between the two groups. The majority of patients in both groups had 6.5 French catheters, though the distribution of sizes trended higher in 5–10 kg patients compared to ≤ 5 kg, with 30.8% and 0%, respectively, having catheters sized above 6.5 French (*P* = 0.008). In our center, circuits were anticoagulated predominantly with prostacyclin (n = 46, 90.2%). The filter life was observed to be longer in children ≤ 5 kg with Median (IQR) life of 52 (35, 71) in comparison to children weighing 5–10 kg of 38 (19, 64) (Table 4). However, there was no difference seen on Kaplan Meier analysis of filter life in the two groups (Log rank analysis *P* = 0.053) (Fig. 1). Blood flow rates were significantly higher in children ≤ 5 kg compared to those weighing between 5-10 kg (Median 14.3 ml/kg/min (IQR 11.4-17.8) versus 8.6 ml/kg/min (7.1-10.8), *P* < 0.01).

### Comparison of survivors and non-survivors

Table 5 shows the baseline characteristics of survivors and non-survivors, and Table 6 compares the nature of CRRT they were administered. There was no statistically significant difference between survivors and non-survivors with respect to sex, indication for CRRT, time spent in PICU prior to CRRT initiation, baseline laboratory tests, vascular access size. Survivors were of significantly of higher weight (Median 6.5 kg vs 4.0 kg, *P* = 0.008) and age (194 days vs 25.5,* P* = 0.027), had longer PICU stay (24 days vs 7 days, *P* < 0.001), longer duration of effective CRRT (120 h vs 64.5 h, p = 0.045), lower PIM2 scores on admission (19.2 vs 55.5, *P* < 0.001), and had lower oxygen requirements (FiO_2_ 52.88% vs 72.62%, *P* = 0.002).

**Table 5:** Characteristics of survivors versus non-survivors

Data	**Survivors (N = 27)**		**Non-survivors (N = 24)**		***P* value**
Male, *n* (%)	15	(55.5)	11	(45.8)	0.579
Age, d^a^	194	(16, 395)	25.5	(9.5, 172.5)	**0.027**
Weight (kg)^a^	6.5	(3.9, 10)	4	(3.3, 5.3)	**0.008**
PICU length of stay, d^a^	24	(13, 33)	7	(2, 12)	** < 0.001**
Hours in PICU prior to CRRT^a^	20	(5, 62.5)	13.5	(6, 41.25)	0.755
Fluid overload > 10% (CRRT 0 h), n (%)	10	(37.04)	12	(50)	0.405
Fluid overload % (CRRT 0 h)^a^	7.17%	(2.47,12.11)	10.01%	(3.99,14.73)	0.190
Vasopressor dependency, *n* (%)	22	(81.48)	17	(70.83)	0.511
PIM2 score @ PICU admission^a^	19.2	(3.9, 45.9)	55.48	(40.08,78.95)	** < 0.001**
Paw (CRRT 0 h)^a^	14	(10, 24)	20	(13, 25.5)	0.296
FiO_2_ (CRRT 0 h)^b^	52.88	± 3.85	72.62	± 5.03	**0.002**

Bold denotes *p*<0.05

^a^Median (IQR 25%, 75%)

^b^Mean (SD)

**Table 6:** Comparison of characteristics of Continuous Renal Replacement Therapy in children according to survival status

Data	**Survivors (N = 27)**		**Non-survivors (N = 24)**		***P*** **value**
Hours of CRRT (Effective)^a^	120	(70, 384)	64.5	(16, 210)	**0.045**
Mean Filter life per Individual (hrs)^b^	43.48	± 20.58	37.25	± 22.92	0.311
CRRT catheter site, n (%)					
Femoral vein (15.6%) Internal jugular vein (76.4%) Subclavian vein (7.84%)	6 21 0	(22.2) (77.7) (0)	2 18 4	(8.3) (75) (16.6)	**0.048**
CRRT catheter size, n (%)					
6F (1.96%) 6.5F (82.35%) 8F (13.73%) 10F (1.96%)	1 21 4 1	(3.7) (77.7) (14.8) (3.7)	0 21 3 0	(87.5) (12.5)	1.000
Blood flow rate (mL/kg/min)^a^	8.80	(7.69, 12.50)	13.6	(7.71, 17.83)	0.054
CRRT dose (mL/kg/h)^a^	60	(51.5, 79)	60	(47, 66)	0.675
Anticoagulation, *n* (%)					
Prostacyclin Heparin	26 1	(96.3) (3.7)	20 0	(83.3) (0)	
No anticoagulant	0	(0)	4	(16.67)	**0.043**
Serum creatinine, μmol/L^a^	51	(27, 170)	55.5	(38,89)	0.25
Serum urea, mmol/L^a^	7.9	(5.4, 13.0)	6.75	(3.8, 17.2)	0.463
Base excess, mEq/L^b^	-5.33	± 7.21	-6.44	± 9.65	0.64
Serum lactate, mmol/L^a^	4.5	(1.5, 7.8)	5.6	(2.61, 10.66)	0.183
Serum bicarbonate, mmol/L^a^	20.90	± 6.43	20.84	± 8.40	0.982
Key indication for CRRT, *n* (%)					
Renal failure ALF High ammonia High lactate Fluid overload > 10% Multiple reasons	19 (70.4) 13 (48.1) 4 (14.8) 9 (33.3) 9 (33.3) 16 (59.3)		15 (62.5) 14 (58.3) 9 (37.5) 10 (41.7) 12 (50.0) 18 (75.0)		0.552 0.467 0.064 0.539 0.227 0.234
Primary diagnosis, n (%)					
Acute liver failure	10 (37.0)		14 (58.3)		0.128
Chronic liver disease/biliary atresia	7 (25.9)		5 (20.8)		0.669
Sepsis	4 (14.8)		8 (33.3)		0.120
Inborn error of metabolism	7 (25.9)		7 (29.2)		0.796
Other	10 (37.0)		10 (41.7)		0.735
*Some patients had multiple primary diagnoses					

Bold denotes *p*<0.05

^a^Median (IQR 25%, 75%)

^b^Mean (SD)

**Fig. 2:**
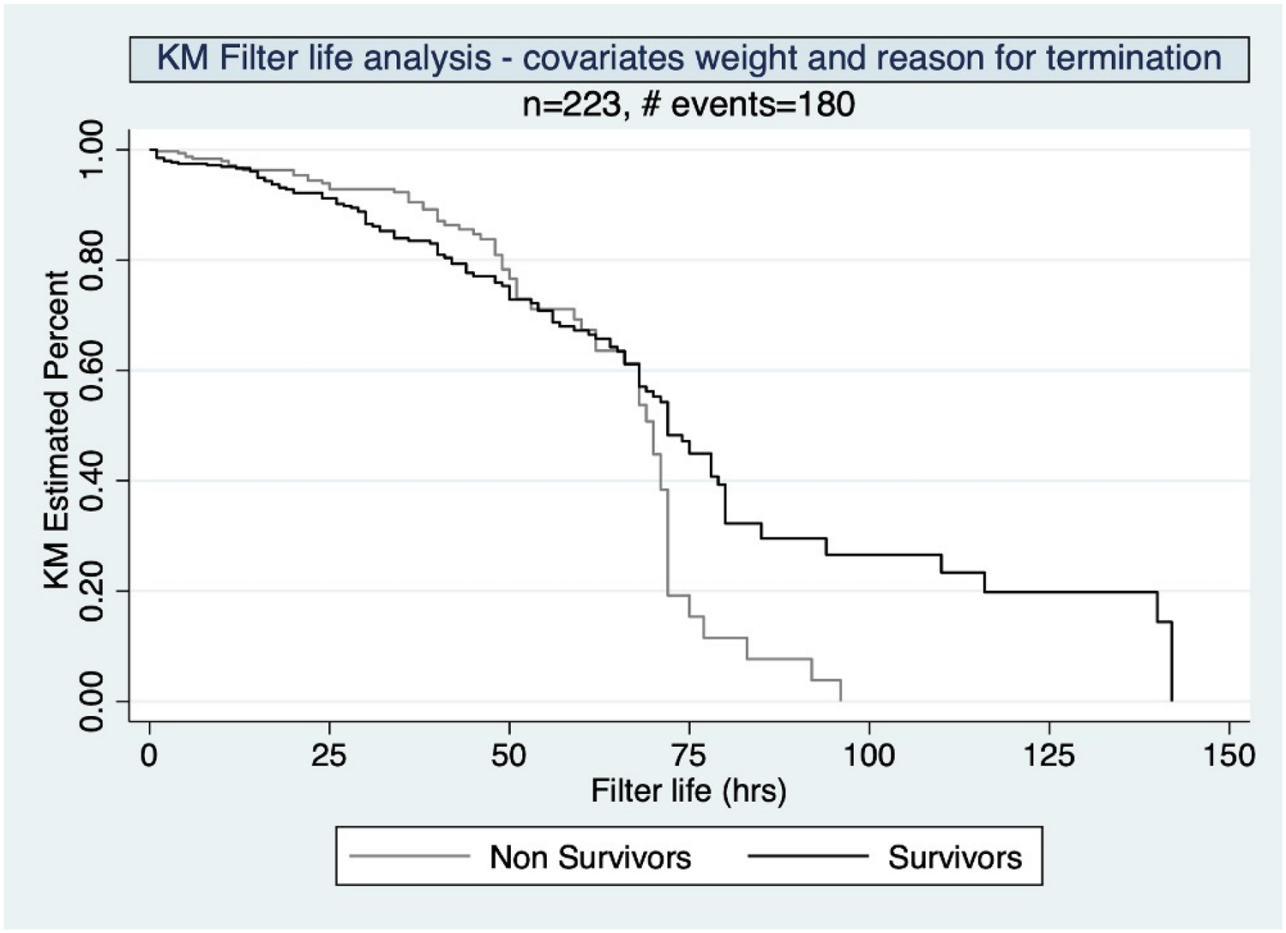
Circuit life analysis in children < 10 kg undergoing CRRT based on Survival, P = 0.1155

There was no significant difference in circuit life in survivors and non survivors on Kaplan Meier analysis (Log rank analysis *P* = 0.1155) (Fig. 2). The effective (*P* = 0.045) and cumulative (*P* = 0.046) duration of filtration, though observed to be different in both survivors and non-survivors (Table 7), did not emerge as predictors after logistic regression analysis.

**Table 7:** Cumulative duration of CRRT in the survivor and the non-survivor group

	**Median (IQR)**		***P*-value**
	**Survivors (n = 27)**	**Non Survivors (n = 24)**	
Hours of CRRT (effective)	120 (70.00, 384.00)	64.50 (16.00, 210.00)	**0.045**
Cumulative CRRT in Hrs	138 (71, 407)	67 (20, 218.5)	**0.046**
CRRT downtime	3 (1, 13)	2.5 (0, 5.5)	0.343

Bold denotes *p*<0.05

### Univariate and multivariate analyses of survival

Table 8 shows univariate analysis for survivors. Patients with lower PIM2 scores, maximum blood flow rates, and FiO2 requirements, had significantly improved survival. Children of older age, higher weight and longer PICU length of stay, were also associated with increased survival. Multivariate regression analysis identified lower PIM2 scores and longer ICU length of stay as independent predictors of survival (Table 9). However, blood flow rate was not an independent predictor of survival.

**Table 8:** Univariate analysis of parameters influencing survival in children undergoing CRRT

	**OR (95% CI)**		***P value***
Age, d	1.004	(1.001, 1.008)	**0.017**
Weight (kg)	1.42	(1.09, 1.83)	**0.007**
PICU length of stay, d	1.06	(1.01, 1.12)	**0.010**
Hours in PICU prior to CRRT	0.99	(0.99, 1.00)	0.935
Fluid overload > 10% (CRRT 0 h)	0.58	(0.19, 1.79)	0.350
Vasopressor dependency	1.80	(0.48, 6.70)	0.376
PIM2 score at PICU admission	0.95	(0.93, 0.98)	**0.0007**
FiO2 (CRRT 0 h)	0.96	(0.93, 0.98)	**0.006**
Renal failure sole indication	0.87	(0.28, 2.69)	0.813
Hours of Effective CRRT	1.001	(0.99, 1.004)	0.236
Male Sex	1.475	(0.48, 4.45)	0.490
Paw at CRRT initiation	0.96	(0.89, 1.03)	0.293
Blood flow rate Max (ml/kg/min)	0.86	(0.75, 0.98)	**0.026**
CRRT Dose (ml/kg/hr)	0.99	(0.96, 1.02)	0.668
Mean Filter Life per individual (hrs)	1.014	(0.988, 1.041)	0.305
Hours of CRRT effective	1.002	(0.999, 1.004)	0.236
Hours of CRRT incl downtime	1.001	(0.999, 1.004)	0.246

Bold denotes *p*<0.05

**Table 9:** Multivariate regression analysis for survival in children < 10 kg undergoing CRRT

	**OR (95% CI)**		***P value***
Age, days	1.01	(0.99, 1.02)	0.121
Weight (kg)	1.00	(0.47, 2.15)	0.985
FiO2 Requirement at CRRT Initiation	0.94	(0.89,1.01)	0.109
PIM2 score at PICU admission	0.89	(0.83, 0.97)	**0.007**
ICU LOS (d)	1.12	(1.00, 1.22)	**0.035**
Blood flow rate (ml/kg/min)	1.00	(0.80, 1.24)	0.980

Bold denotes *p*<0.05

## Discussion

CRRT is a life-saving therapy. Though dedicated neonatal machines are being manufactured with great promise, these are not yet universally available. Consequently, the treating clinician has no choice but to use standard adult-based CRRT machines in smaller children. So, the question remains: are adult-based CRRT machines safe and efficacious in children < 10 kg?

In this retrospective analysis of data over an 8-year period for CRRT in children less than 10 kg, we have shown that CRRT can be utilised in children using adult sized CRRT machines with adaptive adjustments. The proportion of patients who survived in our study (52.9%) is higher than previous studies in children < 10 kg by Symons et al*.* (38%) [19], and Askenazi et al*.* (43%) [9], with the caveat that these involved different machines and cohorts. Our survival rate is similar to that of an earlier case series by Askenazi et al*.* using Aquadex (50%) [20]. Survivors had lower PIM2 scores, which was an independent predictor of survival, lower FiO2 at initiation, and additionally improved survival was seen with increased weight and age. Our study parallels other studies indicating that sepsis and multiorgan failure are common indications for CRRT even in small children. As our centre is a tertiary liver transplant centre, one of the major primary diagnoses in our cohort was acute liver failure, which affirms the use of CRRT as a safe bridging therapy for either spontaneous recovery or liver transplantation [21].

In terms of overall safety and efficacy, the primary aim of this study, this analysis demonstrates that using blood priming routinely for children less than 10 kg did not lead to any significant aberrant complications in terms of electrolyte disturbance or fall in haemoglobin, indicating the safety of blood priming for these children. We found that serum potassium fell by a median of 0.3 mmol/L. This analysis also demonstrates that these machines are effective even in these small children with respect to a fall in serum creatinine and a reduction in fluid overload. These findings should increase clinical confidence in the use of adult machines for these small children.

One of the main challenges in these children is vascular access, and longevity of the circuits. As seen in other studies looking at the causes of circuit change, issues related to vascular access contributed to circuit change—bending or kinking of the Vascath, worsened by the excessive length of the catheter. Interestingly, we found that patients ≤ 5 kg were less likely to have circuit or filter clotting or access issues necessitating a change of filter, and this is important in minimizing treatment interruptions and avoiding blood loss during filter change. This could be explained by the relatively higher blood flow rate in patients ≤ 5 kg.. Another relevant factor is the use of prostacyclin (epoprostenol) as the main anticoagulation for 90% of patients [22]. Prostacyclin has particular value as an anticoagulant in patients with liver disease or a high risk of bleeding, which is relevant as many patients in our cohort had liver disease [22, 23]. A recent retrospective cohort study in adults receiving CRRT demonstrated a trend towards increased survival in the prostacyclin group compared to the heparin group, despite higher SOFA scores in the former group [24].

As described, there are machines which have been specifically designed or adapted for children < 10 kg including CARPEDIEM [25, 26], NIDUS [27, 28], Aquadex, and Japanese Ishikawa. A recent study compared outcomes for children < 10 kg in the US Prospective Pediatric CRRT registry 2001–2005 against those treated with CARPEDIEM in Italy 2013–2018, and found survival to CRRT termination was greater in those treated in the CARPEDIEM registry (97% vs 44%, p < 0.0001) [26]. However, this finding must be interpreted with caution given these registries are maintained in different countries and data were collected over a decade apart. In practical terms, use of different machines for different weight groups within the same program requires rigorous ongoing education and training. The percentage of utility of CRRT machines for CVVH in children < 10 kg in our study is 6.37/year, so having multiple machines may not be cost- and resource-effective. Though our unit used CVVH as the modality of choice, continuous venovenous haemodialysis (CVVH) can also be used with added advantage of smaller vascular catheters, lower blood flow rates and anticoagulation usage.

The main limitation of our study is that this is a retrospective analysis at a single centre. Our PICU is a liver centre, so our findings may not be entirely transferrable to other centres, such as those with a large proportion of patients with cardiac disease, who have a high risk of AKI [29]. Additionally, although we conducted a multivariate analysis, it is difficult to determine associations between survivors and non-survivors due to small numbers and the retrospective observational nature of this study.

## Conclusions

Our in-depth single-center analysis of 51 patients < 10 kg receiving CRRT in line with a consistent protocol demonstrates effective solute clearance, survival rates similar to the existing literature, and with PIM2 scores correlating with survival. No complications were seen related to blood priming and minimal related to vascular access and anticoagulation. Hence CRRT machines used for adults, if used by experienced staff and a size and weight adjusted CRRT prescription, can be safely and effectively used in young children < 10 kg pending universal safe roll-out of neonatal-dedicated CRRT machines.

## Data Availability

The data that support the findings of this study are not openly available but reasonable requests can be made to the corresponding author.
